# Gestational Diabetes Mellitus-Induced Inflammation in the Placenta via IL-1β and Toll-like Receptor Pathways

**DOI:** 10.3390/ijms252111409

**Published:** 2024-10-23

**Authors:** Katarzyna Zgutka, Marta Tkacz, Patrycja Tomasiak, Katarzyna Piotrowska, Przemysław Ustianowski, Andrzej Pawlik, Maciej Tarnowski

**Affiliations:** 1 Department of Physiology in Health Sciences, Faculty of Health Sciences, Pomeranian Medical University, 70-210 Szczecin, Poland; 2 Institute of Physical Culture Sciences, University of Szczecin, 70-453 Szczecin, Poland; 3 Department of Physiology, Pomeranian Medical University, 70-111 Szczecin, Poland; 4 Department of Nursing, Faculty of Health Sciences, Pomeranian Medical University in Szczecin, 70-210 Szczecin, Poland

**Keywords:** gestational diabetes mellitus, GDM, inflammation, interleukin

## Abstract

Gestational diabetes mellitus is characterised by an insufficient insulin response to hyperglycaemia and the development of insulin resistance. This state has adverse effects on the health outcomes of the mother and child. Existing hyperglycaemia triggers a state of inflammation that involves several tissues, including the placenta. In this study, we analysed the putative pathomechanism of GDM, with special emphasis on the role of chronic, sterile, pro-inflammatory pathways. The expression and regulation of the elements of IL-1β and Toll-like receptor (TLR) pathways in GDM maternal blood plasma, healthy placental explants and a choriocarcinoma cell line (BeWo cell line) stimulated with pro-inflammatory factors was evaluated. Our results indicate elevated expression of the IL-1β and TLR pathways in GDM patients. After stimulation with IL-1β or LPS, the placental explants and BeWo cell line showed increased production of pro-inflammatory IL-6, TNFa and IL-1β together with increased expression of the elements of the signalling pathways. The application of selected inhibitors of NF-ĸB, MAPK and recombinant interleukin 1 receptor antagonist (IL1RA) proved the key involvement of the IL-1β pathway and TLRs in the pathogenesis of GDM. Our results show the possible existence of loops of autocrine stimulation and a possible inflammatory pathomechanism in placentas affected by GDM.

## 1. Introduction

During pregnancy, changes in the maternal environment can exert permanent effects on foetal physiology and metabolism and have an impact on the life of the child [1]. One of the most common changes is a state of persistent hyperglycaemia first recognised during pregnancy—gestational diabetes mellitus (GDM). This pathology is defined as glucose intolerance and is characterised as an insufficient insulin response and insulin resistance [1]. The prevalence of GDM is 2.1% to 38% of pregnancies, depending on the region [2].

There are several risk factors related to GDM development: lifestyle factors (obesity, diet, sedentary lifestyle), maternal age, high weight gain during pregnancy, family history of GDM or diabetes mellitus type 2 and the presence of other diseases (i.e., polycystic ovarian syndrome) [3].

During healthy pregnancies, insulin resistance favours nutrient transportation to the developing foetus. Maternal euglycaemia is maintained by changes in the number and activity of pancreatic β-cells, resulting in an elevation in insulin synthesis and release. In GDM, β-cell function is decreased, and insulin levels are not able to compensate for increasing insulin resistance, leading to hyperglycaemia [4]. Next, persistent hyperglycaemia (glucotoxicity) and elevated free fatty acids (lipotoxicity) trigger an inflammatory cascade [5,6]. Inflammation further worsens pancreatic β-cell function and increases insulin resistance in peripheral tissues like muscles, adipose tissue and placenta [7].

In GDM, low-grade, sterile inflammation arises and leads to sequential activation of a variety of inflammatory mechanisms in maternal and gestational tissues [8,9,10]. There are certain molecular pathways that are involved in the inflammatory response, such as the nuclear factor kappa-light-chain-enhancer of activated B cells (NF-κB) pathway, Toll-like receptors (TLRs), peroxisome proliferator-activated receptors (PPARs), sirtuins (SIRTs), phosphoinositide 3-kinases (PI3K), mammalian target of rapamycin kinase (mTOR), 5’AMP-activated protein kinase (AMPK) and the NOD-like receptor family and pyrin domain containing 3 (NLRP3) inflammasome in several tissues, including pancreatic, adipose, muscle and gestational tissues [reviewed in 3].

Several pro-inflammatory cytokines, like TNF-α, IL-1β, IL-6, IL-7, IL-8 and IL-15, were shown to be upregulated in GDM placentas and cord blood [11,12,13,14]. The accumulation of theses pro-inflammatory molecules in placenta causes increased placental weight, a lower foetal-to-placenta weight ratio, an abnormal placenta villi structure and placenta vascularisation, decreased apoptosis, increased autophagy and oxidative stress and mitochondrial damage [14,15,16]. The placenta requires an initial inflammatory response for tissue remodelling and the support of angiogenesis. In GDM, the additional migration of neutrophils and monocytes/macrophages is observed toward the developing placenta, which stimulates pro- and anti-inflammatory responses and may be a cause of the disruption of trophoblast physiology [1].

Since the aetiology of GDM and the activity of particular pro-inflammatory molecular pathways are still under debate, this prompted us to investigate the putative pathomechanism of GDM with a special focus on the IL-1β and TLR pathways. The aim of the present study was to establish a possible role of placenta-derived and placenta-oriented factors involved in sterile inflammation and to show possible approaches to attenuate and block the ongoing loops of inflammatory stimulation in the placenta.

## 2. Results

### 2.1. Expression of IL-1β and TLR Pathway-Related Genes Is Different in GDM Placenta Versus NGT (Normal Glucose-Tolerant) Placenta

**Figure 1:**
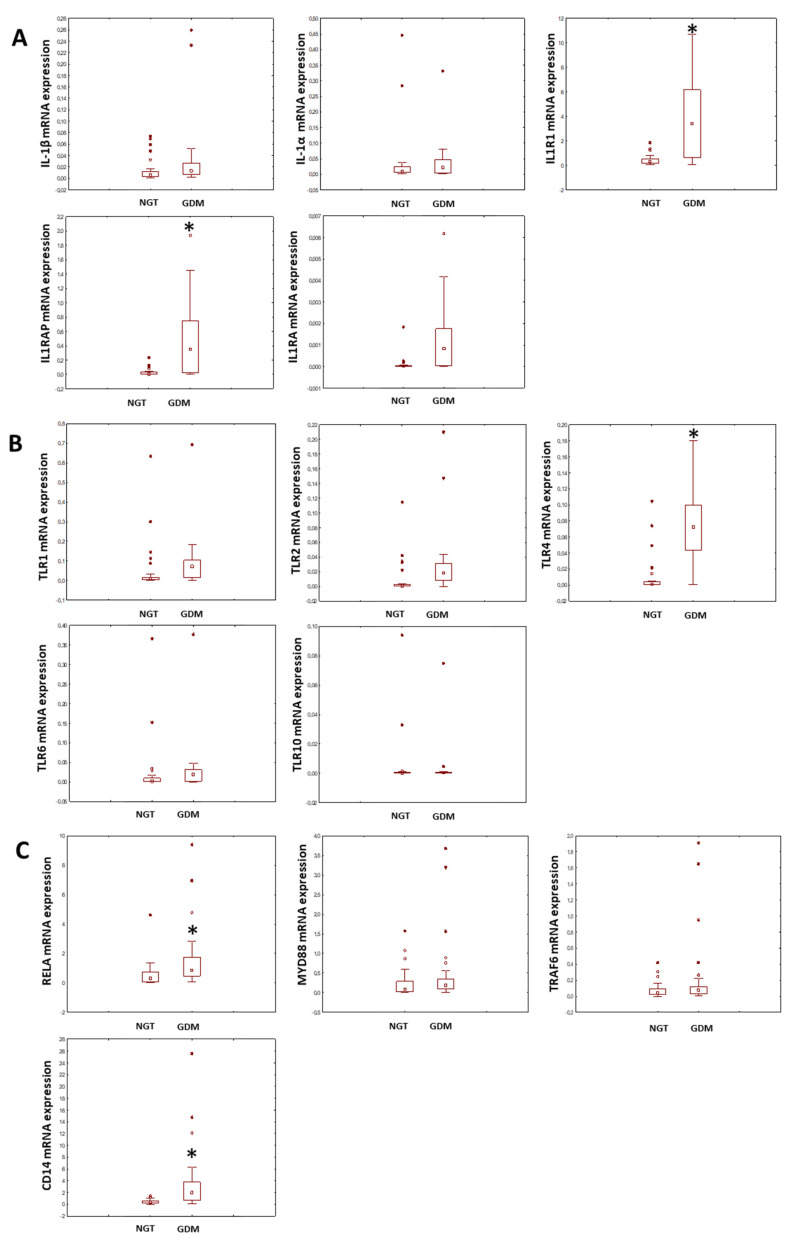
A box and whisker plot showing the absolute mRNA expression of elements of the IL-1β pathway (Panel (**A**)), Toll-like receptors (Panel (**B**)) and elements of the intracellular signal transduction pathway (Panel (**C**)), as assessed by RQ-PCR. The results are presented as the ratio of the gene of interest vs. internal control expression (β2-M; β2-microglobulin); * *p* < 0.05. The dot in the middle of the box represents the median value, and the single dots outside the box represent outliers. NGT (explants from patients with normal glucose tolerance), GDM (explants from patients with gestational diabetes mellitus), IL-1β (interleukin-1β), IL-1α (interleukin-1α), IL1R1 (interleukin 1 receptor), IL1RAP (interleukin 1 receptor accessory protein), IL1RA (interleukin-1 receptor antagonist), TLRs 1, 2, 4, 6, 10 (Toll-like receptors 1, 2, 4, 6, 10), RELA (nuclear factor NF-kappa-B p65 subunit), MYD88 (myeloid differentiation primary response 88), TRAF6 (TNF receptor-associated factor 6) and CD14 (myeloid cell-specific leucine-rich glycoprotein).

In the first attempt to recognise the key elements deregulated in GDM, we analysed the expression of the IL-1β receptor pathway (IL-1 receptor type 1 (IL1R1), IL1 receptor antagonist (IL1RA), IL1 receptor accessory protein (IL1RAP) (Figure 1A)) on placental explants from GDM and normal glucose-tolerant (NGT) women. Additionally, we assessed the expression of Toll-like receptors as the putative elements responsible for the induction and progression of sterile inflammation (Figure 1B) and intracellular elements involved in the transduction of these pro-inflammatory signals (Figure 1C). The choice of genes of interest was based on the available literature.

Among the fifty-four placenta explants used in the experiment, we noted that there were certain, statistically significant, differences in genetic expression. Surprisingly, it was not IL-1β which was overexpressed, but rather, its receptor system, namely IL1R1 (IL-1 receptor) and IL1RAP (IL-1 receptor accessory protein). Of note, we observed an elevation in IL1R1 antagonist expression (IL1RN), but the result was not statistically significant. We also noted significant elevation in TLR4, CD14 and RELA expression in GDM versus NGT placentas.

### 2.2. IL1RA Levels Are Elevated in GDM Peripheral Blood Plasma

**Figure 2:**
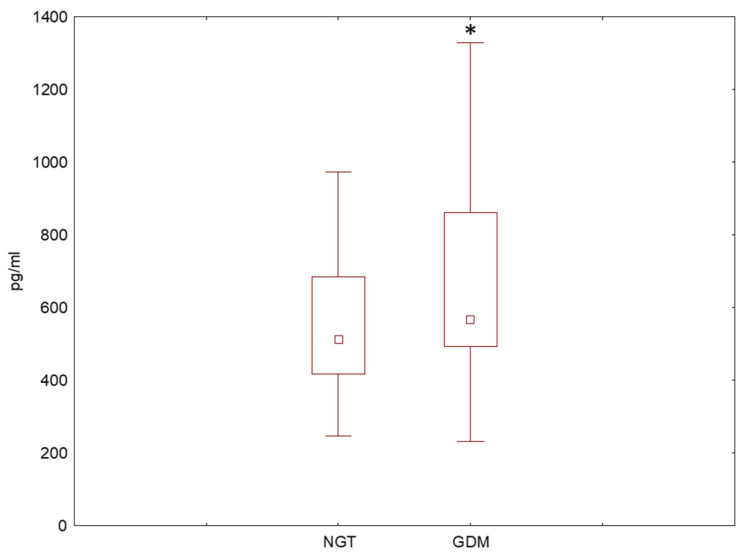
A box and whisker plot showing the **c**oncentration of IL1RA in the peripheral blood plasma of GDM and NGT women as assessed by an immunoenzymatically assay. * *p* < 0.05. The dot in the middle of the box represents the median value. NGT (peripheral blood from patients with normal glucose tolerance), GDM (peripheral blood from patients with gestational diabetes mellitus), IL1RA (interleukin-1 receptor antagonist).

In order to obtain a broader picture of the inflammatory landscape of GDM, we screened peripheral blood for IL-1β and IL1RA levels in GDM patients. When the blood plasma was checked for IL-1β concentration, most samples (both GDM and NGT) were found below the level of detection; however, the concentration of IL1RA was elevated in the peripheral blood of GDM women when compared to healthy controls (Figure 2).

### 2.3. LPS and IL-1β Effectively Produce an Inflammatory Response in Choriocarcinoma Cells and Placental Explants

**Figure 3:**
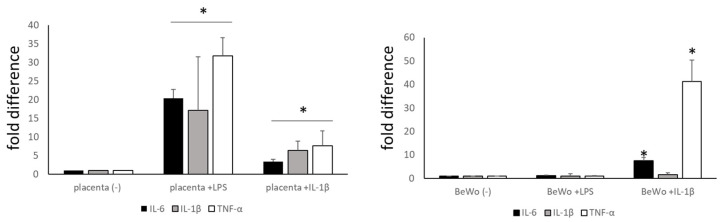
The effects of placental tissue (left) and BeWo cell (right) stimulation by pro-inflammatory molecules, IL1-β (20 ng/mL) and LPS (100 ng/mL) on relative mRNA expression of IL-6, IL-1β and TNF-α. The data are presented as the fold difference, where 1 = unstimulated control samples. * *p* < 0.05 compared with unstimulated controls. All experiments were repeated at least three times with similar results. IL-6 (interleukin 6), IL-1β (interleukin 1β), TNF-α (tumour necrosis factor α), LPS (lipopolysaccharide).

In the next steps of our study, we tried to determine the particular mechanisms triggering the inflammatory response within the placenta. Healthy placental explants were stimulated with lipopolysaccharide (LPS), which is a known activator of the TLR (specifically TLR4) and IL-1β pathways. The explants were stimulated for 20 h and the expression of the typical markers of inflammation (TNF-α, IL-6 and IL-1β) was assessed (Figure 3). In parallel, we performed similar experiments on the choriocarcinoma cell line BeWo, which is commonly used as a model for trophoblast research. We noted significant up-regulation of TNF-α and IL-6 expression induced by LPS and IL-1β, which proves that these molecules are very strong stimulants of the immune response in explants of placenta and the BeWo cell line. IL-1β expression was very low in BeWo cells, and its stimulation was not effective in significantly increasing IL-1β expression. As previously shown, BeWo cells either very poorly or do not respond to LPS stimulation even though they express the TLR4 receptor [17] (Figure 3).

### 2.4. LPS and IL-1β Stimulation Affect Genetic Expression of IL-1β and Toll-like Receptor Pathways

**Figure 4:**
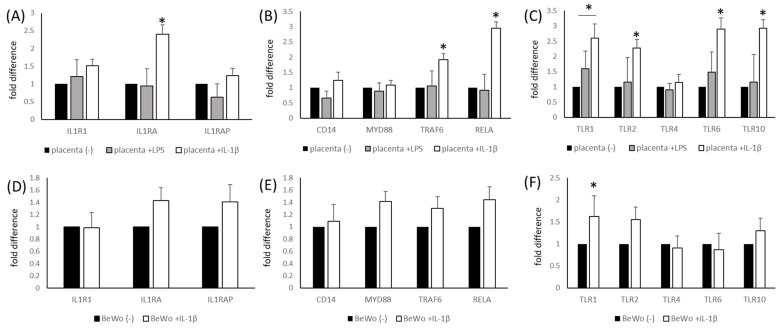
The relative mRNA expression of elements of the IL-1β system (Panels (**A**) and (**D**)), Toll-like receptors (Panels (**C**) and (**F**)) and elements of intracellular signal transduction pathways (Panels (**B**) and (**E**)), as assessed by RQ-PCR in placental tissue (Panels (**A**–**C**)) and BeWo cells (Panels (**D**–**F**)) stimulated by LPS (100 ng/mL) or IL-1β (20 ng/mL). The data are presented as fold differences, where 1 = unstimulated controls. * *p* < 0.05 compared with unstimulated controls. IL-1β (interleukin 1β), IL1R1 (interleukin 1 receptor), IL1RAP (IL1R1 accessory protein), IL1RA (IL1R1 antagonist), TLRs 1, 2, 4, 6 and 10 (Toll-like Receptors 1, 2, 4, 6 and 10), RELA (nuclear factor NF-kappa-B p65 subunit), CD14 (myeloid cell-specific leucine-rich glycoprotein), MYD88 (myeloid differentiation primary response 88), TRAF6 (TNF receptor-associated factor 6) and LPS (lipopolysaccharide).

**Figure 5:**
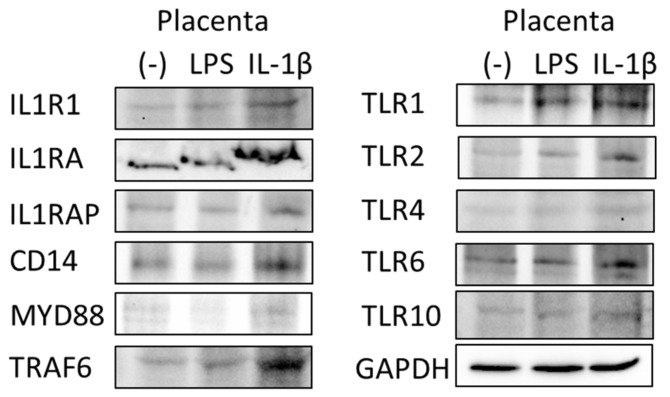
Western blot analysis of changes in protein expression after stimulation with LPS (100 ng/mL) or IL-1β (20 ng/mL). GAPDH (glyceraldehyde-3-phosphate dehydrogenase) served as the loading control. IL-1β (interleukin1β), IL1R1 (interleukin 1 receptor), IL1RAP (interleukin 1 receptor accessory protein), IL1RA (IL1R antagonist), TLRs 1, 2, 4, 6 and 10 (Toll-like receptors 1, 2, 4, 6 and 10), MYD88 (myeloid differentiation primary response 88), TRAF6 (TNF receptor-associated factor 6) and LPS (lipopolysaccharide).

As shown in Figure 4, LPS- and IL-1β-stimulated placental explants and BeWo cells showed the characteristically altered expression of the following elements of the IL-1β receptor pathway: IL-1 receptor type 1 (IL1R1), IL1 receptor antagonist (IL1RA), IL1 receptor accessory protein (IL1RAP), TLRs (TLR1, 2, 4, 6 and 10) and intracellular signal transduction machinery (CD14, MYD88, TRAF6 and RELA). In our experiment, IL-1β stimulation produced the strongest effect, significantly elevating the expression of IL1RA, TRAF6, RELA, TLR1, TLR2, TLR6 and TLR10 in placental explants (Figure 4A–C). We performed a similar experiment on the BeWo cell line, but these cells, as previously mentioned, did not respond to LPS stimulation and showed minimal changes (not statistically significant) in genetic expression; however, the response to IL-1β was significant in TLR1 (Figure 4D–F). These results were recaptured at the protein level in Western blots (Figure 5).

### 2.5. The Application of Inhibitors Attenuates the Pro-Inflammatory Response

**Figure 6:**
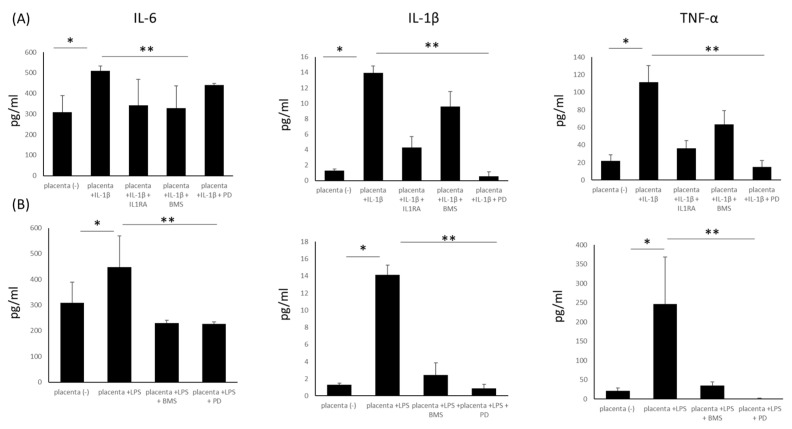
Measurement of pro-inflammatory cytokine concentrations in supernatants collected from placenta explants stimulated with IL-1β (20 ng/mL) (Panel (**A**)) or with LPS (100 ng/mL) (Panel (**B**)) and with selected inhibitors: recombinant IL1RA (100 ng/mL); BMS 345541(12.5 µM)—a highly selective inhibitor of I kappa B kinase that blocks NF-κB-dependent transcription; and a MAPK (mitogen-activated protein *kinase*) inhibitor—PD184352 (5 µM). * *p* < 0.05 compared with unstimulated controls. ** *p* < 0.05 compared with stimulated controls and after the application of inhibitors. IL-1β (interleukin1β), IL-6 (interleukin 6), IL1R (interleukin 1 receptor), LPS (lipopolysaccharide), TNF-α (tumour necrosis factor α), IL1RA (IL1R antagonist).

Next, we wanted to counteract the pro-inflammatory stimulation produced by IL-1β and LPS. Hence, we used certain inhibitors of the tested molecular pathways: recombinant IL1RA; BMS 345541*—*a highly selective inhibitor of I kappa B kinase that blocks NF-κ B-dependent transcription; and a MAPK (mitogen-activated protein kinase) inhibitor—PD184352. Significant downregulation of the inflammatory response after the use of inhibitors at both the protein and mRNA levels (Figure 6 and Figure S1, respectively) was observed. The inhibitors proved their effectiveness; however, they showed variable effects depending on the cytokine produced. In general, and as expected, IL1RA blocked the IL-1β triggered response in placenta explants and BeWo (Figure 6 and Figure S2). Similarly, BMS significantly reduced IL-6 and TNF-α expression and secretion into supernatants collected from placental explants and BeWo cells, but its activity was lower in reducing IL-1β production. The MAPK inhibitor showed the greatest effectiveness in blocking IL-1β and TNF-α secretion and expression (Figure 6, Figures S1 and S2).

**Figure 7:**
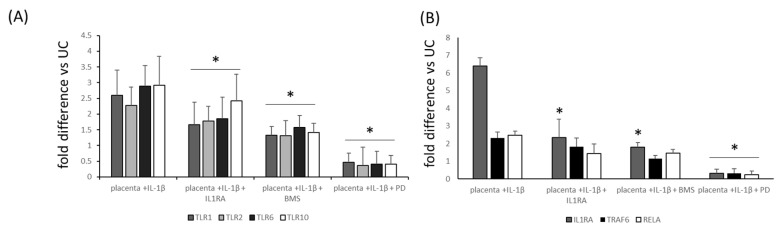
Relative downregulation of mRNA expression of Toll-like receptors 1, 2, 6 and 10 (Panel (**A**)) and IL1RA, TRAF6 and RELA (Panel (**B**)) in placental explants stimulated with IL-1β alone or by IL-1β with selected inhibitors. UC (unstimulated controls), expression = 1. * *p* < 0.05 compared with IL-1β-stimulated samples. TLRs 1, 2, 6, 10 (Toll-like receptors 1, 2, 6, 10), IL-1β (interleukin 1β), IL1RA (IL-1R antagonist), TRAF6 (TNF receptor-associated factor 6), RELA (nuclear factor NF-kappa-B p65 subunit), BMS 345541 *(*a highly selective inhibitor of I kappa B kinase that blocks NF-κB-dependent transcription), PD184352 (an MAPK (mitogen-activated protein kinase) inhibitor).

Finally, we wanted to shed more light on the activity of inhibitors on the expression of the elements of intracellular pathways, so we checked for the most significantly altered proteins in placental explants first stimulated by IL-1β and next blocked by the selected inhibitors (PD184352, BMS 345541 and recombinant IL1RA). As expected, the application of the inhibitors effectively downregulated the expression of IL1RA, TRAF6, RELA, TLR1, TLR2, TLR6 and TLR10 in placental explants, as shown in Figure 7.

## 3. Discussion

Our study focused on the expression and regulation of the elements of IL-1β and Toll-like receptor (TLR) pathways in GDM, with special emphasis on evaluating the possible pathomechanism present in the placenta. Our profiling of placental samples obtained from healthy, glucose-tolerant women and GDM patients revealed the altered expression of genes involved in particular inflammatory processes, specifically the IL-1β pathway and the TLR pathway. We noted significant upregulation of IL1R, IL1RAP, TLR4, RELA and CD14 in placental samples obtained from GDM women. This result was paralleled with increased levels of IL1RA in peripheral blood plasma in GDM women. This observation is symptomatic for the persistent inflammatory state in which multiple pathways are recruited and the expression of multiple molecular components is severely altered [18]. Moreover, our results showed increased expression of the signalling pathways’ participants and increased production of pro-inflammatory molecules (IL-6, TNFa and IL-1β) after stimulation of the placental explants and BeWo cell line with IL-1β or LPS.

Existing hyperglycaemia during GDM, due to insulin insufficiency, is known to cause certain immune dysfunction that leads to leukocyte dysfunction and the increased production and secretion of cytokines, like IL-1β, IL-6 and TNF-α [19,20]. It is known that obesity is one of the most important risk factors for developing GDM, and increased production of pro-inflammatory cytokines in the placenta of obese women is observed [21]. Furthermore, this rising inflammatory status creates so-called sterile inflammation or metaflammation [22,23], which negatively impacts β-cells of the pancreas, adipose and gestational tissues, affecting the structure, function and growth regulation of the placenta [24,25].

The state of chronic, low-grade inflammation is a crucial factor contributing to the development and progression of diabetes. We can imagine that existing gluco- and lipotoxicity induce pro-inflammatory mechanisms involving several para- and autocrine loops of ongoing stimulation. Oxidative and endoplasmic reticulum stress affect the functioning of trophoblasts, monocytes/macrophages and endothelial cells [26,27]. In term GDM placentas, there is an increased number of macrophages and upregulated levels of genetic expression for IL-1β, IL-6, MCP-1, leptin, TNF-α, IL-7, IL-8 and TLR4 [11,13,28]. The accumulation of inflammatory signals causes villous immaturity, villous fibrinoid necrosis, chorangiosis and increased angiogenesis, with an increase in the overall size of the placenta and the deviation of nutrient transport [29]. This uncontrolled, augmented cytokine production in GDM may not only affect the mother’s organs and tissues, but could also compromise the normal development of the growing foetus, with an increased risk of serious complications for the neonate [1].

IL-1β is one of the most central players in the pathogenesis of diabetes. IL-1β signalling is necessary for the inflammatory response, but is highly regulated due to the negative effects of chronic inflammation on body tissues and organs. Even low concentrations of IL-1β are selectively toxic for insulin-producing pancreatic β-cells [5,30]. There have been numerous studies that have related polymorphisms and gene variations in the IL-1β gene with differences in the transcription and expression of the *IL-1β* gene, which could correlate with the development of many autoimmune and inflammatory diseases, such as systemic lupus erythematosus [31], rheumatoid arthritis [32] and multiple sclerosis [33]. In GDM, polymorphisms in the *IL-1β* gene and *IL-1β-*related genes are also intensively investigated [34,35]. Binding to the IL-1 receptor 1 (IL1R1) activates NF-κB pathways, which leads to the increased production and release of other inflammatory mediators, such as TNF-α, IL-6 and IL-1β itself, thus initiating a self-amplifying, autocrine cytokine network [36]. The binding of IL-1β to IL1R1 initiates a cascade of conformational changes in the intracellular proteins, beginning with the recruitment of IL1RAP and further signalling via the adaptor protein MYD88 to the kinases interleukin 1 receptor-associated kinases 2 and 4 (IRAK-2, 4) and the oligomerisation of tumour necrosis factor-associated factor (TRAF) 6. TRAF6 recruits NF-ĸB, p38, Janus kinase and MAPK/ERK to initiate the transcription of hundreds of genes in multiple cell types [37,38]. Importantly, most of these intracellular elements participate in response to other molecules like DAMPs in the Toll-like receptor system [39]. Importantly, NF-kB activation and TLR signalling upregulate the expression and release of pro-IL-1β [40]. The control of IL-1β production and release is precisely regulated and proceeds in two steps, including the initial stimulation of IL-1β expression by a pro-inflammatory signal, with subsequent storage of inactive pro-IL-1β in the cell. The second step involves the production of active, mature IL-1β through the cleavage of its inactive precursor by caspase-1, which is activated in a large cytoplasmic multiprotein complex called the inflammasome [41,42]

IL-1β plays an important role in diabetic pathophysiology, and its levels in peripheral blood serum are significantly elevated in T1DM [43] and T2DM. In GDM, serum levels of IL-1β are also frequently measured; however, its usefulness as a marker of GDM is greatly limited in contrast to serum levels of TNF-α or IL-6 [8,41,44,45,46,47]. The regulation of IL-1β signalling is maintained in healthy individuals but appears to be elevated during chronic pro-inflammatory disease states, which makes this pathway a valuable therapeutic target in diabetes [41]. The master negative regulator of IL-1β signalling is IL1RA. Since it does not induce signal transduction, the levels of production influence its activity. The dysregulation of IL-1β—IL1RA balance is reported to be one of the factors affecting symptoms, as seen in the course of many diseases with an inflammatory component [48,49]. It was shown that ILRA serum levels are elevated in pathologies as diverse as sepsis, cancer, metabolic diseases and autoimmune diseases [50,51,52,53,54]. Since the discovery of the central role of IL-1β in the pathogenesis of type 2 diabetes, an increasing number of studies have investigated the role of IL-1β blockade in insulin resistance and type 2 diabetes. To date, several independent clinical studies have been conducted with a recombinant IL1RA, known as anakinra, or neutralising anti-IL-1β antibodies (gevokizumab, canakizumab and LY21891020). They have demonstrated beneficial effects on metabolic parameters, reducing glycated haemoglobin (HbA1c), increasing insulin sensitivity and β-cell secretion, together with an improvement in inflammatory markers, in patients with T2DM [55,56] and with a history of myocardial infarction [57,58,59,60,61,62,63].

We can speculate that the increase in circulating IL1RA levels corresponds to a delayed event in response to IL-1β production and may represent a preventive mechanism in a long-acting and/or an excessive inflammatory response. In our study, we noted increased IL1RA serum levels and its placental expression in GDM patients; its expression was elevated after the stimulation of healthy placenta with LPS or IL-1β, which is interesting. We are aware of a study by Katra et al. [64] which, in contrast, shows decreased levels of IL1RA in GDM patients; the explanation of this discrepancy in the results may come from various timings of serum collection during the course of the pregnancy.

In our experiments, we have shown that LPS and IL-1β induce an inflammatory response in the form of increased expression and the secretion of IL-1β, IL-6 and TNF-α in healthy placenta explants. In previous reports, it was shown that placenta, adipose tissue and muscle tissue are sensitive to LPS stimulation and react through the increased release of TNF-α and IL-6 [65]. In the case of IL-1β, we observe an autocrine loop that may be present in many cellular contexts, i.e., platelets [66], monocytes [66] or epithelial cells [67]. We demonstrate that the application of inhibitors such as recombinant IL1RA; BMS 345541, a highly selective inhibitor of I kappa B kinase that blocks NF-kappa B-dependent transcription; and the MAPK (mitogen-activated protein kinase) inhibitor PD184352 is effective in attenuating the inflammatory response in the placenta and in BeWo cells. Even though the choriocarcinoma cell line does not characterise IL-1β transcription, the subsequent IL-6 release stimulation with IL-1β and blocking the effect with recombinant IL1RA shows an important mechanism in placental physiology [68].

Toll like receptors are involved in recognising DAMPs. Upon activation, the signalling cascade progresses through the MYD88/TRAF6/NF-κB route, eliciting the expression of further elements of the innate immune response [69]. Of the ten functional TLRs in the placenta [70], TLR4 is the best studied. It is expressed in various tissues, including vascular endothelial cells and the placenta [71]. In our set of experiments, we detected increased expression of the Toll-like receptors TLR1, TLR2, TLR6 and TLR10; their expression was subject to change after stimulation with LPS and IL-1β. The inhibition of NF-kappa B-dependent transcription was effective in regulating TLR receptor expression. TLRs are established elements of the inflammatory milieu in GDM. GDM increases the expression of the TLR4, MYD88 and NF-κB signalling pathways in the placenta [14,72]. To date, the expression of other TLRs in GDM placenta has not been evaluated. Nonetheless, multiple elements of the TLR receptor system are deregulated in GDM to some extent, as shown at the mRNA level in peripheral blood mononuclear cells [73] and amnion membrane biopsies [74]. Our results indicate that there is substantial overlap between important inflammatory pathways of IL-1β/TLR. The formation of this reciprocal activation is a key element of existing sterile inflammation. This state may be resolved upon delivery, but the health complications directly resulting from GDM will have a severe impact on both mother and child. Hence, further research efforts and molecular studies are needed on potential interventions and anti-inflammatory therapies in GDM.

## 4. Materials and Methods

### 4.1. Blood Samples

Fifty-four pregnant women were included in this study. The GDM diagnosis was based on the criteria of the International Association of Diabetes and Pregnancy Study Groups (IADPSG) [75]. Oral glucose tolerance tests (75 g) were performed between weeks 24 and 28 of gestation. A GDM diagnosis required a fasting glucose level of 92 mg/dL (5.1 mmol/L), and the 1 h and 2 h plasma glucose levels had to exceed 180 mg/dL (10.0 mmol/L) and 153 mg/dL (8.5 mmol/L), respectively. Ultimately, 26 patients with GDM treated at the Department of Obstetrics and Gynaecology, Pomeranian Medical University, Szczecin, Poland, were included in this study, as described previously in [76]. The control group comprised 28 pregnant women with normal glucose tolerance (NGT). To be included in this study, pregnancy must have been achieved through natural conception. Patients with chronic infections, autoimmune and inflammatory disorders, neoplastic diseases, type 1 diabetes mellitus (T1DM) and type 2 diabetes mellitus (T2DM), and those experiencing acute or chronic diabetes complications were excluded from this study. Written consent for participation was obtained from each included patient. This study was reviewed and approved by the local Ethics Committee of Pomeranian Medical University, Szczecin, Poland (KB-0012/40/14).

### 4.2. Placental Samples

Human placentas were obtained from healthy women (with normal glucose tolerance) and women with GDM, immediately after spontaneous vaginal delivery after 37 weeks of gestation. After the removal of amniotic membranes, soft tissues of the placenta from the maternal side were dissected, washed and minced into approximately 1–2 mm diameter pieces. The explants from GDM patients were placed into 2 mL cryotubes with 1.5 mL RNAlater (Invitrogen, Carlsbad, CA, USA) and stored at −80 °C for future analysis. Explants from healthy women were cultured in Dulbecco’s modified Eagle’s minimum essential medium (DMEM) supplemented with L-glutamine (2 mM), 100 IU/mL penicillin, 10 µg/mL streptomycin (Sigma, St Louis, MO, USA) and 10% heat-inactivated foetal bovine serum—FBS (Life Technologies, Carlsbad, CA, USA).

### 4.3. Placental Stimulation

Immediately after isolation, the explants were stimulated for 20 h in different combinations. We used 10% FBS medium as a negative control. Explants were stimulated for 20 h with LPS (lipopolysaccharides from Escherichia coli O55: B5) (100 ng/mL) alone; with LPS (100 ng/mL) and BMS 345541 (4(2′-aminoethyl)amino-1,8-dimethylimidazo(1,2-a)quinoxaline) (12.5 µM); with LPS (100 ng/mL) and PD184352 (2-[(2-chloro-4-iodophenyl)amino]-N-cyclopropylmethoxy)-3,4 difluorobenzamide) (5 µM); with IL-1β (20 ng/mL) alone or with the inhibitors; and with IL-1β (20 ng/mL) and IL1RA (100 ng/mL) (Sigma, St Louis, MO, USA). At the end point of the incubation, the supernatant was collected and frozen at −80 °C until analysis. The explants were divided into two parts for later RNA and protein extraction after homogenization with an ultrasonic homogenizer (UP50H, Hielscher Ultrasound Technology, Teltow, DE, USA). The first group of placental samples were placed into a 2 mL cryotubes with 1.5 mL RNAlater (Invitrogen, Carlsbad, CA, USA) and stored at −80 °C. The second group of samples were placed in 2 mL cryotubes with 1.5 mL radioimmunoprecipitation assay (RIPA) lysis buffer (Santa Cruz Biotech, Santa Cruz, CA, USA).

### 4.4. Culture of BeWo Cells Line

Human trophoblast BeWo cells (CL-98) were purchased from the American Type Culture Collection (ATCC, Manassas, VA, USA). The cells were kept in culture flasks on F-12K Medium (Kaighn’s Modification of Ham’s F-12 Medium) supplemented with 100 IU/mL penicillin, 100 μg/mL streptomycin (Sigma, St Louis, MO, USA) and 10% foetal bovine serum (FBS) (Life Technologies) in a humidified incubator at 37 °C and 5% CO_2_. BeWo cells were separately added to 24-well plates (3 × 10^4^/1000 µL/well) in F-12K Medium with 10% FBS at 37 °C and 5% CO_2_. After 24 h in culture, the cells were stimulated according to the scheme described above for placental explants. The timing of the stimulation was based on a time–response experiment, and the optimal time point was chosen. After stimulation, the supernatant was collected and frozen at −80 °C for future analysis. Cells were also collected for later RNA and protein extraction.

### 4.5. Extraction of Protein and Analysis with Western Blotting

Cellular proteins from homogenates were extracted with radioimmunoprecipitation assay (RIPA) lysis buffer (Santa Cruz Biotech, Santa Cruz, CA, USA) containing a protease inhibitor cocktail (Roche, Basel, Switzerland) and a phosphatase inhibitor (Roche). After the determination of protein concentration with a Bradford assay, the standard protocol of Western blotting was followed to determine the protein abundance in placental explants. Briefly, 20–30 μg protein was electrophoresed in 12% sodium dodecyl-sulfate (SDS)-polyacrylamide gel and transferred to a nitrocellulose membrane (BioRad, Hercules, CA, USA). After blocking with 5% BSA in TTBS, the membrane was probed with primary antibodies (anti-GAPDH- loading control (Abcam, ab181602, Cambridge, UK), anti-IL1R1 (Abcam, ab106278), anti-IL1RA (Abcam, ab124962), Anti-IL1RAP (Abcam, ab232950), anti-CD14 (Abcam, ab183322), anti-MYD88 (Abcam, ab133739), anti-TRAF6 (Abcam, ab33915), anti-TLR1 (Abcam, ab68158), anti-TLR2 (Abcam, ab68159), anti-TLR4 (Abcam, ab13556), anti-TLR6 (Abcam, ab37072) and anti-TLR10 (Abcam, ab228511)) overnight at 4 °C. After washing the membranes in TTBS, the following appropriate secondary antibodies were used: goat anti-rabbit IgG H&L (Abcam, ab205718), goat anti-mouse IgG H&L (Abcam, ab205719) and rabbit anti-mouse IgG H&L (Abcam, ab6728). Protein visualisation was performed using a Novel ECL Chemiluminescent Substrate Reagent Kit (Invitrogen, Carlsbad, CA, USA).

### 4.6. ELISA

The concentration of the selected proteins, IL1RA, IL-1β, IL-6 and TNF-α, was measured using the immunoenzymatic ELISA method using commercial test kits (R&D, Minneapolis, MN, USA). The procedure was performed according to the manufacturer’s recommendations. Absorbance readings wereperformed using an Infinite^®^ 200 PRO plate reader (Tecan, Mannedorf, Switzerland) at a wavelength of 450 nm, and the concentrations of IL1RA, IL-1β, IL-6 and TNF-α were expressed in pg/mL.

### 4.7. Real-Time Quantitative Reverse Transcription PCR (RQ-PCR)

Total RNA was isolated from BeWo cells and placental explants with an RNeasy Kit (Qiagen, Valencia, CA, USA). Total RNA was extracted from the homogenates using an RNeasy Fibrous Tissue Mini Kit (Qiagen, Hilden, Germany) in accordance with the manufacturer’s protocol. A Lambda Bio+ spectrophotometer (PerkinElmer, Waltham, MA, USA) was used to determine the RNA concentration and purity. RNA was reverse-transcribed with MultiScribe reverse transcriptase and oligo(dT) primers (Applied Biosystems, Foster City, CA, USA). A quantitative assessment of mRNA levels was performed via real-time reverse transcriptase polymerase chain reaction (RT-PCR) on an ABI 7500 Fast instrument using Power SyBR Green PCR Master Mix reagent (Applied Biosystems, Foster City, CA, USA) and selected primers (IBB, Warsaw, Poland). β2-microglubuluin was used as a reference gene to normalise the messenger RNA (mRNA) levels in the different samples [77,78,79]. Each 20 µL reaction contained 2 μL of diluted cDNA. Two technical replicates were used for each sample; the mean cycle threshold (CT) values and the 2−ΔCt method were used for the calculation of absolute expression, and 2−ΔΔCt method was used for calculation of relative expression (fold difference). The primer sequences are available in Supplementary Table S1.

### 4.8. Statistical Analysis

The Mann–Whitney U-test was used to compare the gene expression results between the GDM and NGT groups. The results are presented as the mean ± standard error of the mean (SEM). In the in vitro experiments, statistical data analysis was performed using the non-parametric Mann–Whitney or Student’s *t*-test, with *p* < 0.05 considered significant. The statistical analysis was conducted using Statistica 13 software (StatSoft, Kraków, Poland). The results are presented as mean values and standard deviation (SD). *p* < 0.05 was considered statistically significant.

## 5. Conclusions

Overall, our study showed the important dysregulation of crucial inflammatory pathways in GDM placentas. We noted that GDM placentas are characterised by elevated expression and the high activation of both receptors and signal transduction elements of the IL-1β and TLR pathways. In order to shed some light on the possible mechanisms driving the inflammatory reactions, we performed a series of stimulation-based experiments showing the very high inductive potential of IL-1β and LPS, and their involvement in the creation of autocrine loops of ongoing inflammatory activation. The inhibition of these pathways may be pivotal following proper control in preventing gestational diabetes and its consequences for the mother and newborn.

## Data Availability

The data presented in this study are available upon request from the corresponding author.

## Supplementary Materials

The following supporting information can be downloaded at: https://www.mdpi.com/article/10.3390/ijms252111409/s1.
