# Supplementary material for: Gestational Diabetes Mellitus-Induced Inflammation in the Placenta via IL-1β and Toll-like Receptor Pathways
Source: Int J Mol Sci. 2024 Oct 23;25(21):11409. doi: 10.3390/ijms252111409 (PMC11546908; doi:10.3390/ijms252111409)
Supplement: Supplementary file 1 [file ijms-25-11409-s001.zip › ijms-3248404-supplementary.pdf]

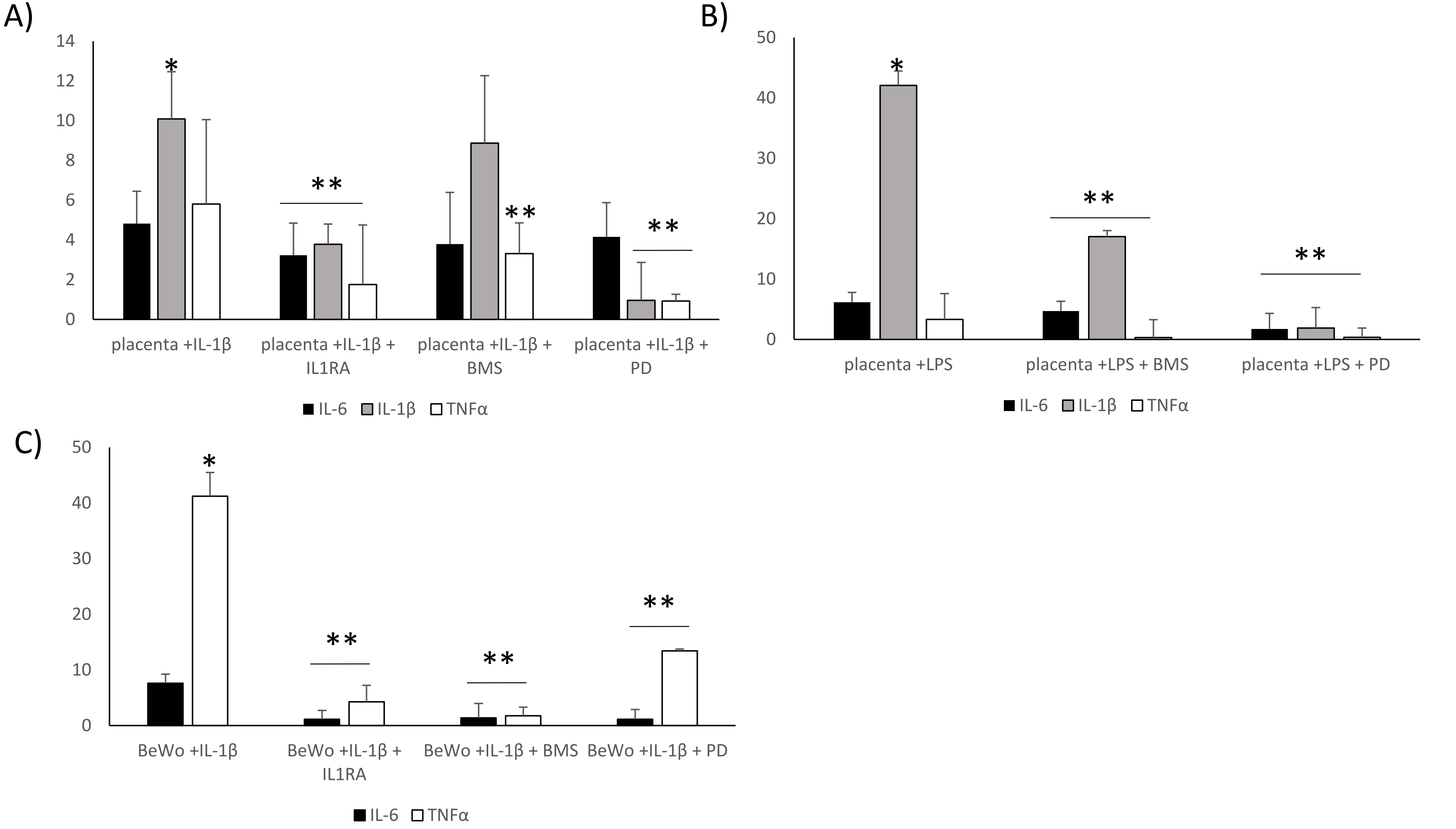

Figure S1. RQ-PCR measurement of pro-inflammatory cytokine expression in the placenta explants stimulated with IL-1β (20ng/mL) (Panel A) or with LPS (100ng/mL) (Panel B), and in BeWo cell line (Panel C) and with selected inhibitors: recombinant IL1RA (100ng/mL), *BMS* 345541(12,5µM) - a highly selective inhibitor of I kappa B kinase that blocks NF-κB-dependent transcription, and MAPK (mitogen-activated protein *kinase*) inhibitor – PD184352 (5µM). * $p<0.05$ as compared with stimulated controls. ** $p<0.05$ as compared with stimulated controls and after application of inhibitors. IL-1β (interleukin1β), IL-6 (interleukin 6), IL1R (interleukin 1 receptor), LPS (lipopolysaccharide), TNF-*α* (tumour necrosis factor *α*), IL1RA (IL1R antagonist).

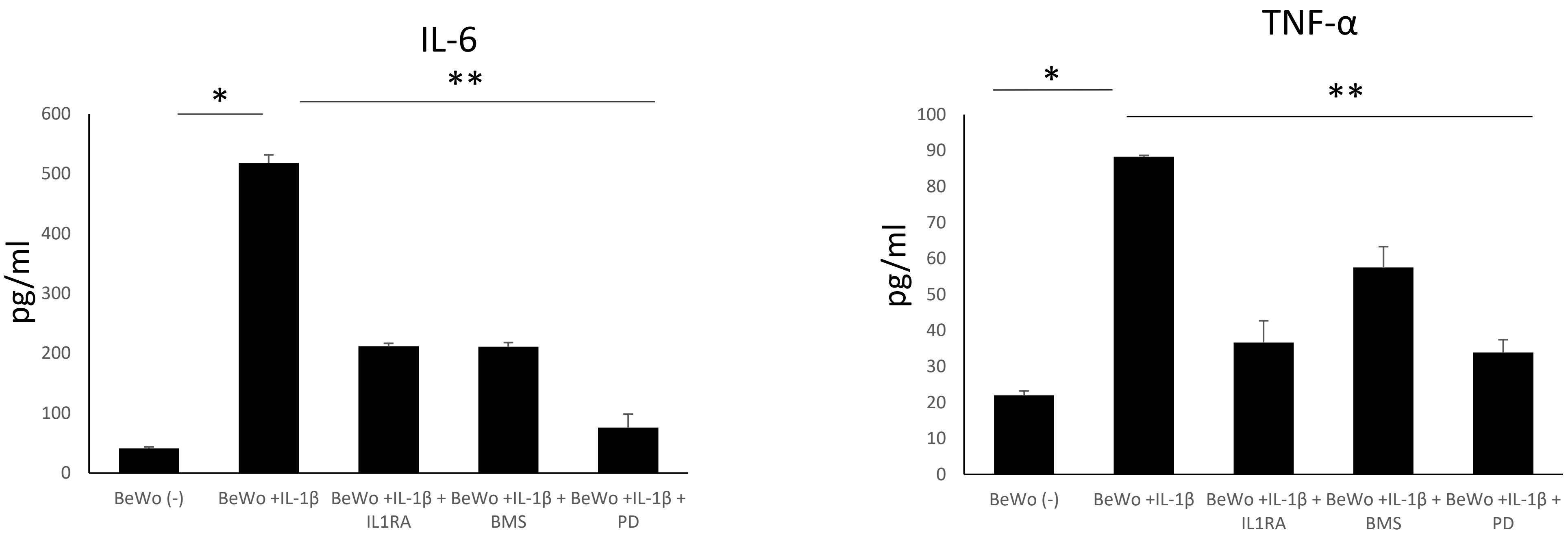

Figure S2. ELISA measurement of pro-inflammatory cytokine concentrations (IL-6 and TNF-*α*) in supernatants collected from BeWo cell line stimulated with IL-1β (20ng/mL) or with LPS (100ng/mL) and with selected inhibitors: recombinant IL1RA (100ng/mL), *BMS* 345541(12,5μM) - a highly selective inhibitor of I kappa B kinase that blocks NF-κB-dependent transcription, and MAPK (mitogen-activated protein *kinase*) inhibitor – PD184352 (5μM). * $p<0.05$ as compared with unstimulated controls. ** $p<0.05$ as compared with stimulated controls and after application of inhibitors. IL-1β (interleukin1β), IL-6 (interleukin 6), IL1R (interleukin 1 receptor), LPS (lipopolysaccharide), TNF-*α* (tumour necrosis factor *α*), IL1RA (IL1R antagonist expression).

Supplementary Table S1. Primer sequences used in the study.

Name:	Sequence
BMG_F	AATGCGGCATCTTCAAACCT
BMG_R	TGACTTTGTCACAGCCCAAGATA
TNFA_R	GAGGACCTGGGAGTAGATGAG
TNFA_F	CCTCTCTCTAATCAGCCCTCTG
IL6_R	GGTTGTTTTCTGCCAGTGCC
IL6_F	AATTCGGTACATCCTCGACGG
IL1B_F	AATCTGTACCTGTCCTGCGTGTT
IL1B_R	TGGGTAATTTTTGGGATCTACACTCT
IL1R1_F	ATGAAATTGATGTTCGTCCCTGT
IL1R1_R	ACCACGCAATAGTAATGTCCTG
IL1a_F	AGATGCCTGAGATACCCAAAACC
IL1a_R	CCAAGCACACCCAGTAGTCT
IL1RAP_F	ACACTTCTGTGGTGTGTAGTGA
IL1RAP_R	TGGTGTCTAGTCCCCAGTCAT
TLR1_F	AGCTGCCAGAAGATGAGGTC
TLR1_R	AATCAGGCCAGCCCTCTAAC
TLR2_F	ATCCTCCAATCAGGCTTCTCT
TLR2_R	GGACAGGTCAAGGCTTTTTACA
TLR4_F	CTGCAGGTGCTGGATTTATCC
TLR4_R	GGTGGCTTAGGCTCTGATATGC
TLR6_F	GCCCAAACCTGTGGAATATC
TLR6_R	ACACGGTGTACAAAGCTGTC
TLR10_F	CATGGCCAGAAACTGTGGTC
TLR10_R	CATCCAGGGAGATCAGTTAG
MYD88_F	ACTGCTCGAGCTGCTTACCAA
MYD88_R	CTCCTGCTGCTGCTTCAAGAT
RELA_F	CCCATCTTTGACAATCGTGC
RELA_R	CTGGTCCCGTGAAATACACC
TRAF6_F	ATGCGGCCATAGGTTCTGC
TRAF6_R	TCCTCAAGATGTCTCAGTTCCAT
CD14_F	ACGCCAGAACCTTGTGAGC
CD14_R	GCATGGATCTCCACCTCTACTG
IL1Ra_F	CATTGAGCCTCATGCTCTGTT
IL1Ra_R	CACTGTCTGAGCGGATGAA
